# Performance of the Modified Mini-Mental State Examination (3MS) in Assessing Specific Cognitive Function in Patients Undergoing Peritoneal Dialysis

**DOI:** 10.1371/journal.pone.0166470

**Published:** 2016-12-02

**Authors:** Yi Li, Xue Tian, Zu-Ying Xiong, Jin-Lan Liao, Li Hao, Gui-Ling Liu, Ye-Ping Ren, Qin Wang, Li-Ping Duan, Zhao-Xia Zheng, Wen-Xiang Quan, Jie Dong

**Affiliations:** 1 Renal Division, Department of Medicine, Peking University First Hospital; Beijing, China; 2 Renal Division, Peking University Shenzhen Hospital, Shenzhen, China; 3 Renal Division, the Second Hospital of Anhui Medical University, Anhui, China; 4 Renal Division, the Second Affiliated Hospital of Harbin Medical University, Heilongjiang, China; 5 Renal Division, Handan Central Hospital, Hebei, China; 6 Peking University Sixth Hospital; Peking University Institute of Mental Health; Key Laboratory of Mental Health, Ministry of Health, Beijing, China; Postgraduate Medical Institute, INDIA

## Abstract

**Purpose:**

While Cognitive impairment (CI) has been identified as an independent risk factors for mortality in patients undergoing peritoneal dialysis (PD), it is inadequately assessed. We evaluated the applicability of the Modified Mini-Mental State Examination (3MS) in assessing specific cognitive function and compared it to a detailed neuropsychological test battery as the reference standard.

**Methods:**

In this multicentric cross-sectional study, we enrolled 445 clinically stable patients from five PD units, who were undergoing PD for at least 3 months. The 3MS was evaluated for general cognitive function. A detailed neuropsychological battery including domains of immediate memory, delayed memory, executive function, language, and visuospatial ability were evaluated as reference standards. Sensitivity and specificity of the 3MS was determined by using receiver operating characteristic (ROC) analysis.

**Results:**

The CI prevalence evaluated by 3MS was 23.6%. PD patients with CI performed worse in all cognitive domains. The 3MS correlated well with specific cognitive domains. However, 18.5%, 57.4%, 12.6%, 8.8%, and 41.2% of patients whom were idendified as normal by 3MS still showed executive dysfunction, immediate memory impairment, delayed memory impairment, and language-ability and visuospatial-ability impairment, respectively. The 3MS identified patients having specific cognitive dysfunction with varied extent of diagnostic value, with 0.50, 0.42, 0.35, 0.34, and 0.26 of Youden index in executive function, delayed memory, language ability, immediate memory, and visuospatial ability, respectively.

**Conclusions:**

The 3MS is not a comprehensive instrument for major cognitive domains in PD patients. It could, however, be used for executive dysfunction and delayed memory impairment screening.

## Introduction

Peritoneal dialysis (PD) as home-care therapy requires patients to self-monitor and self-manage their treatment [1] and, therefore, is partly dependent on cognitive function [2]. Cognitive impairment (CI) has been shown to be an independent predictor of mortality [3, 4] and dialysis withdrawal [5] in patients undergoing dialysis. Unfortunately, the prevalence of general CI is high, ranging between 27% and 67% among patients with end-stage renal disease [3, 6–8]. Patients treated with hemodialysis perform particularly worse in orientation and attention and executive function[9]. Early detection of CI and porformance in specific cognitive domains in these population is thus critical.

The Mini-Mental State Examination (MMSE) is a well-established tool for screening the CI in general population and in patients with chronic kidney disease [10, 11]. Morever, the Modified Mini-Mental State Examination (3MS) with extra questions and an extended scoring system has been reported superior to MMSE with higher sensitivity and specificity, in diagnosing CI and dementia [12–14]. The 3MS takes only 15–20 min to administer and is likely to be widely adopted as a screening tool. However, data regarding the suitability of 3MS compared to a detailed neuropsychological test battery to assess major cognitive domains such as executive, memory, language, and visuospatial ability in a dialyzed population is rather limited. Most recently, a single study in hemodialysis (HD) patients showed that individuals with normal scores of MMSE still endured varied extents of memory dysfunction and executive impairment [15].

In this study, we aimed to evaluate the applicability of 3MS compared to a detailed neuropsychological test battery as reference for assessing specific cognitive function based on a multicentric cross-sectional dataset. The ability to distinguish between PD patients with and without specific CI across the major cognitive domains was examined by assessing the sensitivity and specificity and the Youden index of the 3MS.

## Results

### Basic characteristics and CI

A total of 667 patients were eligible for the study and 495 (74.2%) gave consent, with 445 (89.8%) completing all laboratory analysis and detailed cognitive testing. The mean age, gender, dialysis duration and primary kidney diseases were not significantly different between participants included and excluded. The basic data of our participants was in accordance with the general characteristics of the PD population in China [16]. The mean ages were 51.3 years, and PD durations were 25.1 months. Of these 455 patients, 52.4% were men, 22.7% diabetics, 20.7% had a history of CVD, and 51.9% were educated to high school or higher levels. The prevalence of CI as diagnosed by 3MS was 23.6% in PD patients (**Table 1**). As for major cognitive domains, the highest prevalence was 64.5% for immediate memory impairment, followed by visuospatial-ability impairment (47%), executive dysfunction (29.9%), delayed memory impairment (20.7%), and language-ability impairment (14.2%).

**Table 1:** Clinical characteristics of PD patients with and without CI as diagnosed by 3MS.

	Total	CI group	No CI group	*P*
	(n = 445)	(n = 105)	(n = 340)	
**Age (years)**	51.3±14.2	57.5±12.2	49.4±14.3	<0.001
**Male (%)**	233 (52.4%)	45(42.9%)	188(55.3%)	0.026
**PD duration (months)**	25.1(11.1–49.1)	24.0(9.5–47.7)	25.9(11.1–49.6)	0.442
**Primary kidney disease**				
*Diabetic nephropathy (%)*	89(20.%)	29(27.8%)	60(17.8%)	
*Hypertensive nephropathy (%)*	59 (13.4%)	19(18.2%)	40(11.8%)	
*Chronic glomerulonephritis (%)*	206 (46.7%)	39(37.5%)	107(31.7%)	
*Other (%)*	87 (19.7%)	17(16.3%)	70(20.7%)	
**DM (%)**	101 (22.7%)	35(33.3%)	66(19.4%)	0.003
**Cardiovascular disease (%)**	92 (20.7%)	31(29.5%)	61(17.9%)	0.01
**Charlson Index**	5 (3–8)	5 (3–10)	4(3–8)	0.021
**Level of education**				<0.001
*Elementary school or lower*	78 (17.5%)	50(47.6%)	28(8.2%)	
*Middle school*	136 (30.6%)	26(24.8%)	110(32.4%)	
*High school*	129 (29.0%)	23(21.9%)	106(31.2%)	
*Above high school*	102 (22.9%)	6(5.7%)	96(28.2%)	
**Body mass index (kg/m**^**2**^**)**	22.8±3.5	23.5±3.5	22.6±3.48	0.025
**Systolic blood pressure (mmHg)**	135.2±18.3	137.9±17.6	134.5±18.4	0.101
**Diastolic blood pressure (mmHg)**	82.1±12.3	79.6±10.9	82.7±12.6	0.046
**Hemoglobin (g/L)**	105.1±17.8	103.1±19.4	105.7±17.2	0.208
**Serum albumin (g/L)**	36.0±5.6	33.6±5.6	36.7±5.4	<0.001
**Triglyceride (mmol/L)**	1.9±1.3	2.1±1.5	1.9±1.2	0.254
**Total cholesterol (mmol/L)**	4.7±1.1	5.1±1.1	4.6±1.1	0.002
**Serum sodium (mmol/l)**	139.2±3.1	138.7±3.7	139.4±2.8	0.082
**Calcium (mmol/l)**	2.4±0.2	2.3±0.2	2.4±0.2	0.927
**Phosphate (mmol/l)**	1.7±0.4	1.6±0.5	1.7±0.4	0.198
**hsCRP (mg/L)**	2.9(0.9–8.6)	4.3(1.4–12.7)	2.5 (0.8–7.1)	<0.001
**RRF (ml/min)**	2.1 (0.0–5.4)	2.4(0.0–5.2)	2.0(0.00–5.62)	0.981
**Total Kt/V**	1.9 (1.7–2.1)	1.8(1.6–2.1)	1.9 (1.7–2.1)	0.284
**Total Ccr (ml/min/1.73m**^**2**^**/week)**	54.0 (46.2–65.1)	54.9 (45.9–65.7)	53.8 (46.2–64.6)	0.724

Abbreviations: CI: cognitive impairment; PD: peritoneal dialysis; DM: diabetes mellitus; hsCRP: high-sensitivity C-reactive protein; RRF: residual renal function; Kt/V, urea clearance per week; Ccr: creatinine clearance per week.

As compared to patients with normal cognitive function, those with CI tended to be older, of female gender, and less educated. They also had a greater burden of DM, CVD, and CVD risk factors such as higher body mass index, increased cholesterol, and inflammation reflected by higher CRP and lower serum albumin levels, but were more likely to have lower diastolic blood pressure (*p* < 0.05 for all) (**Table 1**).

### Correlations of CI as diagnosed by 3MS and major cognitive domains by a detailed neuropsychological test battery

We further divided all subjects into two groups according to the presence or absence of CI. Patients in the CI group had significantly longer completion time on Trail A and Trail B, and lower scores of immediate and delayed memory, and language and visuospatial ability as assessed by the detailed neuropsychological test battery (*P* < 0.001 for all, **Table 2**). Furthermore, Spearman correlation analysis indicated that the scores of 3MS were significantly negatively associated with completion time on Trail A and Trail B, but positively associated with scores of immediate and delayed memory, language ability, and visuospatial ability (*P* < 0.001 for all, **Table 3**).

**Table 2:** Scores of 3MS and major cognitive domains by a detailed neuropsychological test battery between PD patients with and without CI as diagnosed by 3MS.

	Total	CI group	No CI group	*P*
	(n = 445)	(n = 105)	(n = 340)	
3MS (scores)	88(80–93)	71(65–75)	91(86–94)	<0.001
Trail A (seconds)	65(47–92)	107(78–168)	59(44–92)	<0.001
Trail B (seconds)	151(106–230)	262(183–473)	130(100–190)	<0.001
Executive dysfunction (n, %)	133 (29.9%)	70 (66.7%)	63 (18.5%)	<0.001
Immediate memory (scores)	76(61–85)	57(49–67)	78(69–90)	<0.001
Immediate memory impairment (%)	287 (64.5%)	92 (87.6%)	195 (57.4%)	<0.001
Delayed memory (scores)	94(81–100)	78(60–90)	96(86–102)	<0.001
Delayed memory impairment (%)	92 (20.7%)	49 (47.1%)	43 (12.6%)	<0.001
Language ability (scores)	95(86–101)	85(76–91)	96(90–104)	<0.001
Language ability impairment (%)	63 (14.2%)	33 (31.4%)	30 (8.8%)	<0.001
Visuospatial ability impairment (scores)	84(64–105)	64(58–92)	87(69–108)	<0.001
Visuospatial ability impairment (%)	209 (47%)	69 (65.7%)	140 (41.2%)	<0.001

Abbreviations: 3MS, modified mini-mental state examination; CI, cognitive impairment; PD, peritoneal dialysis.

**Table 3:** Correlation coefficients between scores of 3MS and major cognitive domains by a detailed neuropsychological test battery.

	3MS	Trail A	Trail B	Immediate memory	Delayed memory	Language ability	Visuospatial ability
3MS	1	-0.563*	-0.58*	0.627*	0.576*	0.534*	0.419*
Trail A	-0.563*	1	0.771 *	-0.412 *	-0.350 *	-0.342 *	-0.287 *
Trail B	-0.580*	0.771 *	1	-0.426*	-0.381*	-0.355*	-0.286*
Immediate memory	0.627*	-0.412*	-0.426*	1	0.637*	0.454*	0.333*
Delayed memory	0.576*	-0.350*	-0.381*	0.637*	1	0.461*	0.519*
Language ability	0.534*	-0.342*	-0.355*	0.454*	0.461*	1	0.230*
Visuospatial ability	0.419*	-0.287*	-0.286*	0.333*	0.519*	0.230*	1

*P < 0.001 for all.

**Fig 1:**
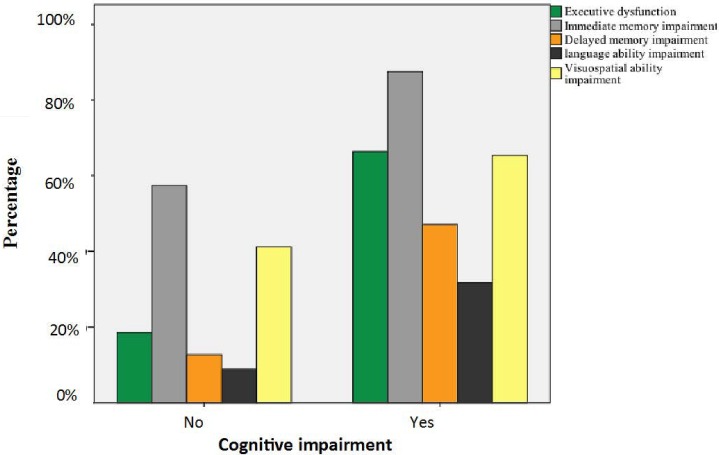
The percentage of major cognitive dysfunction in patients undergoing peritoneal dialysis (PD) with and without cognitive impairment (CI) diagnosed by the modified mini-mental state examination (3MS).

However, when major cognitive domains were considered as the reference standards, 33.7%, 12.5%, 52.9%, 68.3%, and 34.6% of patients with CI were not found to have executive dysfunction, immediate memory impairment, delayed memory impairment, and language-ability and visuospatial-ability impairment, respectively, based on 3MS (i.e., false negative rate). By contrast, 18.5%, 57.4%, 12.6%, 8.8%, and 41.2% of patients without CI (i.e., false positive rate) had executive dysfunction, immediate memory impairment, delayed memory impairment, and language-ability and visuospatial-ability impairment, respectively (**Fig 1**).

### ROC analysis and predictive values

The ROC curves illustrated different capacities of 3MS, displaying its individual sensitivity, specificity, and AUC, while major cognitive domains were considered as reference methods, respectively (**Table 4**). The ROC analysis displayed an optimal cut-off of the 3MS at ≤82 points for executive dysfunction, ≤88 for immediate memory impairment, ≤82 for delayed memory impairment, ≤80 for language-ablity impairment and ≤89 for visuospatial ability impairment. The sensitivity, specificity and an AUC of each optimal cut-off value were also shown in Table 4. The Youden indices of 3MS achieved the highest in 0.50 for executive dysfunction, followed by 0.42 for delayed memory impairment, 0.35 for language-ability impairment, 0.34 for immediate memory impairment, and 0.26 for visuospatial-ability impairment.

**Table 4:** Criterion (cut-off*) values and coordinates of the ROC curves of 3MS and major cognitive domains by a detailed neuropsychological test battery.

Criterion of 3MS	Sensitivity (95%CI)	Specificity (95%CI)	AUC	Youden Index
	Executive dysfunction			
≤81	61.59(52.9–69.7)	85.63(81.3–89.2)		0.50
≤82	67.39(58.9–75.1)	82.57(78.0–86.5)	0.742	
≤83	71.01(62.7–78.4)	78.29(73.4–82.6)		
	Immediate memory impairment			
≤87	61.02(55.4–66.5)	71.34(63.8–78.1)		0.34
≤88	65.50(59.9–70.8)	68.90(61.2–75.9)	0.662	
≤89	69.01(63.6–74.1)	64.02(56.2–71.4)		
	Delayed memory impairment			
≤81	61.46(51.0–71.2)	80.82(75.8–84.3)		0.42
≤82	65.62(55.2–75.0)	76.39(71.7–80.7)	0.753	
≤83	68.75(58.5–77.8)	71.94(67.0–76.5)		
	Language ability impairment			
≤79	54.05(42.1–65.7)	79.26(75.0–83.1)		0.35
≤80	56.76(44.7–68.2)	77.78(73.4–81.7)	0.717	
≤81	58.11(46.1–69.5)	75.56(71.1–79.7)		
	Visuospatial ability impairment			
≤88	67.57(61.0–73.7)	57.56(51.0–63.9)		0.26
≤89	72.07(65.7–77.9)	53.78(47.2–60.2)	0.672	
≤90	74.77(68.5–80.3)	48.83(41.8–54.9)		

*Optimal cut-off score based on maximal sensitivity and specificity using the receiver operating characteristic (ROC) analysis.

Abbreviation: AUC, area under the curve.

## Discussion

In this large-scale, multicentric cohort of Chinese PD patients, we found that the prevalence of general CI diagnosed by 3MS was 23.6%. As compared to previous data, the overal CI prevalence in our PD subjects was very close to that in their HD counterparts as diagnosed by the MMSE [15, 17, 18] or a computing mean values of z-scores for all cognitive domains [19]. Our PD patients had more severe impairment in immediate memory (two-third) and less impairment in executive dysfunction (one-third) than the HD population [7, 15, 20], supporting the US multicentric cross-sectional data [7]. Similarly, CVD history and CVD risk factors also contributed to the occurrence of CI in the PD population [15, 21–23].

The main goal of this study was to determine the discriminating value of 3MS in the PD population across major cognitive domains. From the previous studies, the reliability of 3MS has been shown to be higher than the MMSE in individuals with different educational backgrounds, normal community elders, geriatric rehabilitation patients, and dementia cases. [13, 14]. However, for PD patients, although the 3MS were closely associated with major cognitive domains, the false positive and negative rate of 3MS was also remarkably high as shown in this study. A further ROC analysis showed that 3MS could distinguish the CI with the best value on executive dysfunction, followed by delayed memory, language-ability impairment, immediate memory impairment, and visuospatial-ability impairment, with the detailed neuropsychological test battery as the reference gold standardmethods. Some items of the present 3MS version are relevant to executive function, which was designed to detect common dementia subtype early, in which executive function is most prominently affected [24].

Taking into account that the detailed neuropsychological test battery is a complicated and time-consuming tool in clinical practice, our findings also revealed that specific cut-off scores for different domains of cognitive function. The optimal cut-off scores based on the maximal sensitivity and specificity using the ROC analysis were 82, 88, 82, 80, and 89 of the 3MS in discriminating executive dysfunction, immediate memory, delayed memory, and language- and visuospatial-ability impairment. Combined with above findings, we recommended 3MS to be a screening tool of executive dysfunction and delayed memory with the cut-off value of 82, due to the relatively good sensitivity and specificity in these two domains. However, our findings are not conclusive regarding a score higher than 80, the present criterion for CI by 3MS, as being normal for immediate memory and visuospatial ability in PD patients.

Taken together, our findings indicate that the 3MS could not be considered as a comprehensive tool for assessment of specific cognitive function, especially on immediate memory and visuospatial impairment. Notably, the process of PD therapy itself would be influenced by cognitive dysfunction in various aspects. Executive and memory functions broadly encompass processes responsible for diet management, fluid restrictions, blood pressure and glucose self-monitoring, and compliance to complex medication regimens. Language ability is related to communication with the PD staff, and helps to make decisions on critical medical issues. Visuospatial ability plays an important role in bag exchange, exit-site care, and catheter protection. All these domains are critical to the quality of PD therapy. Therefore, the best option in clinical practice is to simultaneously examine the general and specific cognitive function in order to identify underlying CI. Moreover, we also need to explore whether impairment in specific cognitive domains result in inverse clinical outcomes such as CVD events, PD-related infection, psychological problems, and poor quality of life.

Several strengths of this study should be noted. First, this is one of the largest multicentric studies to examine the performance of comprehensive cognitive domains in PD patients. Secondly, we further suggested the discriminating values of 3MS, and the optimal cut-off score of 3MS used for assessment of special cognition function by using a comprehensive, multidimensional spectrum of cognitive neuropsychological tests as the reference. This information would be helpful in screening specific cognitive impairment, especially on executive and delayed memory function. In addition, all assessments of cognitive function were performed in a separate room with one medical staff to one patient, and relatively less variability in cognitive function suspected for the stable PD treatment, ensure the reliability of our findings.

Our study also has some limitations. First, although the domains of cognition based on neuropsychological battery were applied, it is possible that it was not adequately sensitive and comprehensive to measure subtle cognitive changes in patients with milder CI. However, the RBANS is the best reference tool for cognitive function for our population, as its reliability and validity have been already proved in the Shanghai and Beijing Chinese populations [25, 26]. Secondly, the general and specific cognitive function was only examined one time. Repeat measurements over a longer period would be better and more effective to determine the discriminating values of 3MS. However, only a minority of patients exhibited significant individual cognitive fluctuations as shown in the previous HD study [20]. Finally, because all our patients were Chinese speaking, the generalizability of our results is somewhat limited.

In conclusion, 3MS is a limited tool for detecting comprehensive cognitive performance. Our results alert clinicians to highlight the special domains of cognitive function despite a normal general test like the 3MS. However, the 3MS could be applied for screening the executive dysfunction and delayed memory impairment owing to its relatively good discriminating values for these two domains. On the other hand, as detailed neuropsychological test battery while being a gold standard might still be quite impractical in most settings. More practical, comprehensive and well-validated screening tools for CI also need to be developed with respect to clinical and academic applications.

## Methods

### Study design and participants

This is the affiliated study of a multi-center cross-sectional survy on cognitive funciton in PD. The detailed study design was published elsewhere[27]. Five PD centers from five provinces (Beijing, Heilongjiang, Hebei, Anhui, and Guangdong) located at four geographical regions (north, northwest, east, and south) in China participated in this multicentric cross-sectional study. All these centers have professional doctors and nurses dedicated to PD. Data from each center were collected within a strict quality control framework, further inspected, and optimized to ensure the integrity and accuracy of the database. All study investigators and staff members completed a training program that taught them the methods and processes of the study. A manual of detailed instructions for data collection was distributed. The ethics committee of Peking University First Hospital approved the study. Patients gave written consent for their information to be stored in the hospital database and for subsequent research use.

This study enrolled prevalent PD patients between March 2013 and March 2014. Inclusion criteria for participants were as follows: patients aged ≥ 18 years; undergoing PD for ≥3 months and clinically stable; able to undergo all measurements and respond to questionnaires as required. Patients were excluded if they had a systemic infection, acute cardiovascular events, active hepatitis or cancer, surgery or trauma in the month prior to the study, and all other study-obstructive conditions such as severe eyesight loss, language incompatibility, illiteracy, mental disturbance (e.g., preexisting dementia or confusion, other mental disorders) and upper-limb disability. We defined the preexisting dementia, confusion and any other mental disorders according to ICD-10[28] criterion by the evaluation of psychologist. All the subjects received conventional glucose-based, lactate-buffered PD solutions (Ultrabag; Baxter Healthcare, Guangzhou, China). Patients’ data were collected and were used in this study between May 2013 and May 2014. Authors have no access to information that could identify individual participants during or after data collection.

### Clinical characteristics

Demographics and comorbidities were recorded, including age, gender, education level, durations of PD, body mass index, systolic and diastolic blood pressure, primary kidney disease, the presence of diabetes mellitus (DM), and history of cardiovascular disease (CVD). Mean arterial pressure was calculated. Level of education was recorded as the highest school level at which a diploma was received, that is, elementary school or lower; middle school; high school; or above high school. CVD was recorded if one of the following conditions was present: angina; class III–IV congestive heart failure (NYHA guidelines); transient ischemic attack; and history of myocardial infarction, cerebrovascular accident, and peripheral arterial disease [29]. Cerebrovascular accident including was recorded separately.

### Laboratory methods

After overnight fasting while continuing PD therapy, venous blood samples were drawn from all patients for biochemical measurements. Serum levels of sodium, albumin, calcium, phosphate, triglycerides, total cholesterol, high-sensitivity C-reactive protein (hsCRP), and hemoglobin were calculated as the mean of measurements taken over the preceding 3 months. Biochemical profiles were investigated using an automatic Hitachi chemistry analyzer. Residual renal function (RRF) was defined as the mean of residual creatinine and urea clearance from a 24-h urine collection. Dialysis adequacy was defined as total Kt/V and creatinine clearance.

### Cognitive function

The 3MS [14] was applied to test overall cognitive function. Global cognitive impairment was defined as a score of less than 80 in the 3MS test in previously observational studies of cognitive function [8, 30–32]. Because mean scores on the 3MS vary by education, we used a 3MS cut-off point of <75 for individuals with less than a high school education and <80 for individuals with a high school education [31].

A detailed neuropsychological test battery was evaluated as the reference gold standard. Executive function including decision-making and processing speed was assessed by Trail making tests A (Trail A) and B (Trail B) [33]. In addition, subtests of Repeatable Battery for the Assessment of Neuropsychological Status (RBANS) were adopted to assess immediate memory (list learning and story memory); delayed memory (list recall, list recognition, story recall, and figure recall); visuospatial skill (figure copy); and language ability (picture naming and semantic fluency) respectively [34]. The raw scores were transferred to age-standardized T-score for all subtests of RBANS. The reliability and validity of RBANS has previously been proved in the Shanghai and Beijing population [25, 26]. Executive dysfunction was defined as a Trail A score of more than 75 s and Trail B score of more than 180 s [35–37]. T scores less than 1 SD below the published mean in education-grouped Chinese population were identified as impaired for the immediate, delay memory, language and visuospatial function. [38].

Assessments of cognitive function were performed in a separate room with one medical staff to one patient. Totally four medical staff participated in this study as observers, and they all completed a training program of the methods employed to ensure the integrity and accuracy of the assessment.

### Statistical analysis

Continuous data were presented as means ± SD. Durations of PD, Charlson Comorbidity Index, RRF, and hsCRP were presented as medians with interquartile ranges, owing to high data skewness. Categorical variables were presented as proportions. One-way analysis of variance, Kruskal–Wallis, or the Chi-square tests were used to compare the intergroup differences in demographic and biochemistry data.

Correlations of scores of 3MS and completion time of Trail A and Trail B, and scores of immediate memory, delayed memory, language, and visuospatial ability were examined by using Spearman’s rank correlation coefficient analysis. The ability of the 3MS to screen for specific CI in PD patients was evaluated through the comparison to the abovementioned major cognitive domains. To establish the discriminative validity of the 3MS in identifying patients with and without specific cognitive dysfunction, sensitivity and specificity levels for 3MS scores at varying cut-off values were evaluated using the receiver operating curve (ROC) analysis. Area under the curve (AUC) was calculated to ascertain the quality of 3MS scores as a diagnostic tool for specific cognitive function. An AUC of 0.5 is no better than expected by chance, whereas a value of 1.0 signifies a perfect tool. The optimal timepoint of 3MS cut-off value was defined as the earliest point that provided significant discrimination between patients with and with without specific CI on a cognitive domain with optimal sensitivity, specificity, and Youden index.

All probabilities were two-tailed, and the level of significance was set at 0.05. Odds ratios and 95% confidence intervals were calculated. Statistical analysis was performed by using SPSS for Windows, software version 20.0 (IBM, New York, US), and Medcalc for Windows software version 9.2.1.0 (Medcalc software, Broekstraat, Belgium).

## Supporting Information

S1 File STROBE checklist. (DOCX)
